# Women and the Medical Societies

**Published:** 1899-03-25

**Authors:** 


### WOMEN AND THE MEDICAL SOCIETIES.

The Ophthalmological Society has resolved to admit women to its membership. It is to be congratulated on its decision. We can quite understand the feeling that existed in many quarters against admitting women to the medical profession. It was a feeling with which we could have no sympathy, but it was one for which much could be said by those who felt that way. When, however, women had once been admitted full members of the profession there seemed but small excuse for keeping them out of the medical societies. If, as every one admits to be the case, the societies are useful agencies in maintaining a high standard of medical ethics, there seems no reason why their good influence should not be available for the benefit of medical women as well as of medical men.

